# Impact of anatomical reverse remodelling in the design of optimal quadripolar pacing leads: A computational study

**DOI:** 10.1016/j.compbiomed.2021.105073

**Published:** 2022-01

**Authors:** Cristobal Rodero, Marina Strocchi, Angela W.C. Lee, Christopher A. Rinaldi, Edward J. Vigmond, Gernot Plank, Pablo Lamata, Steven A. Niederer

**Affiliations:** a Cardiac Electro-Mechanics Research Group, Biomedical Engineering Department, King ´s College London, London, United Kingdom; b King's College London, Interdisciplinary Medical Imaging Group, London, United Kingdom; c Institute of Electrophysiology and Heart Modeling, Foundation Bordeaux University, Bordeaux, France; d Bordeaux Institute of Mathematics, UMR-5251, University of Bordeaux, Bordeaux, France; e Medical University of Graz, Gottfried Schatz Research Center - Biophysics, Graz, Austria

**Keywords:** Multipoint pacing, Multipolar pacing, Lead optimization, CRT, Virtual cohort, Digital twin

## Abstract

Lead position is an important factor in determining response to Cardiac Resynchronization Therapy (CRT) in dyssynchronous heart failure (HF) patients. Multipoint pacing (MPP) enables pacing from multiple electrodes within the same lead, improving the potential outcome for patients.

Virtual quadripolar lead designs were evaluated by simulating pacing from all combinations of 1 and 2 electrodes along the lead in each virtual patient from cohorts of HF (n = 24) and simulated reverse remodelled (RR, n = 20) patients. Electrical synchrony was assessed by the time 90% of the ventricular myocardium is activated (AT090). Optimal 1 and 2 electrode pacing configurations for AT090 were combined to identify the 4-electrode lead design that maximised benefits across all patients.

LV pacing in the HF cohort in all possible single and double electrode locations reduced AT090 by 14.48 ± 5.01 ms (11.92 ± 3.51%). The major determinant of reduction in activation time was patient anatomy. Pacing with a single optimal lead design reduced AT090 more in the HF cohort than the RR cohort (12.68 ± 3.29% vs 10.81 ± 2.34%).

Pacing with a single combined HF and RR population-optimised lead design achieves electrical resynchronization with near equivalence to personalised lead designs both in HF and RR anatomies. These findings suggest that although lead configurations have to be tailored to each patient, a single optimal lead design is sufficient to obtain near-optimal results across most patients. This study shows the potential of virtual clinical trials as tools to compare existing and explore new lead designs.

## Highlights

• A common optimal multi-pole lead design delivers a similar reduction in activation times with CRT compared to personalised lead designs.
• As the heart remodels the benefit of CRT on the reduction in activation time decreases.
• This study shows the potential of virtual clinical trials as tools to explore new pacing lead designs.

## Abbreviations

CRT: Cardiac Resynchronization Therapy
RV: Right ventricle
LV: Left ventricle
HF: Heart failure
MPP: Multipoint pacing
RR: Reverse remodelled
UVC: Universal Ventricular Coordinates
IN: Inferior
IL: Infero-lateral
LA: Lateral
AL: Antero-lateral
AN: Anterior
CV: Conduction velocity
FEC: Fast endocardial conduction
TAT: Total activation time
AT090: Time taken to activate 90% of the volume of the biventricular myocardium
HAC: Hierarchical Agglomerative Clustering

## 1 Introduction

Cardiac Resynchronization Therapy (CRT), where the right ventricle (RV) and left ventricle (LV) are paced to return the heart to a synchronous contraction, is one of the main treatments for dyssynchronous heart failure (HF) [1,2]. However, up to 40% of patients do not show an improvement (non-responders) [3,4]. One of the main factors that determines CRT success is the location of the pacing electrode in the LV free wall [5,6]. To address this issue, multipoint pacing (MPP) or quadripolar leads, that allow up to four pacing locations to be evaluated from a single lead position, have been developed [7].

Quadripolar leads, now in routine use, can be programmed to avoid phrenic nerve stimulation, improve reverse remodelling [8,9], reduce radiation exposure [10] and increase lead stability. These improvements do not increase the procedural time of the intervention nor do they reduce implant success rates [10]. Quadripolar leads are provided by multiple vendors and come in multiple forms with different spacing between the electrodes [7]. While there are many leads to choose from, it is not clear which lead configurations will achieve the best response in most patients nor if different configurations will be better in patients with different severities of HF.

Answering these questions is particularly challenging in experimental or clinical set-ups due to wide variability between the patients’ anatomy and function. One of the options to cover this variability is with more lead designs and configurations, but it is still not feasible to try all the designs in an in-vivo or bench scenario. Techniques such as network meta-analysis could be useful in these cases [11,12], but the number of studies and sample sizes are still insufficient. However, advances in cardiac computer simulations now provide platforms for performing *in-silico* clinical studies [13] using virtual patient cohorts [14] where the full design space can be evaluated across multiple patient-specific models. Moreover, using healthy anatomies can serve as a representation of a reverse remodelled (RR) anatomy, where the heart changes back to a healthier phenotype when responding to CRT.

In this paper, 24 patient-specific biventricular computational models of HF patients were used to answer three main questions. First, to determine the single optimal quadripolar lead design for achieving synchronous ventricular activation across all HF anatomies in different potential LV vein locations. Second, to estimate the benefit of tailoring the lead design in each patient and lead location within HF anatomies. Third, 20 patient-specific biventricular computational models of healthy subjects were used to approximate RR anatomies, to test if the optimal lead design changes in cases where the heart responds to CRT.

## 2 Methods

### 2.1 Virtual cohorts

**Fig. 1:**
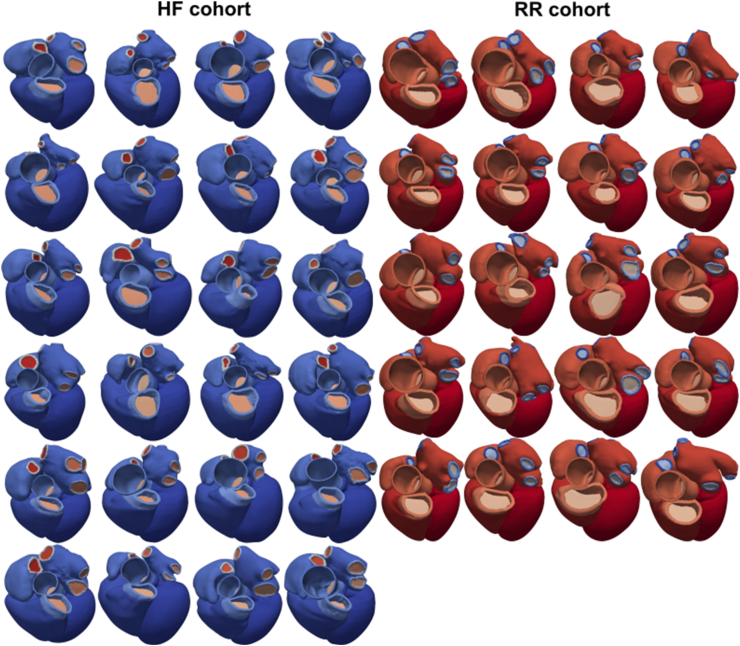
Four-chamber heart meshes from 24 HF patients (blue, left) and 20 RR (red, right) CT datasets. Only the ventricles were used in this study. (For interpretation of the references to colour in this figure legend, the reader is referred to the Web version of this article.)

A publicly available virtual cohort of 24 patients who met guideline indications and received CRT was used to model HF patients, as presented by Strocchi et al. [15]. Neither CT nor MRI imaging was available for reverse remodelled CRT cases. However, when CRT is effective, patients revert to near normal phenotype [8,9]. For this reason, a publicly available virtual cohort of 20 healthy (asymptomatic) individuals [16] was used as approximations for ideally reverse remodelled (RR). These cohorts will be referred to as HF and RR, respectively and as RR + HF when treated together as a single cohort. Subjects in the HF and RR cohorts were 67 ± 14 and 51 ± 8 years old, with 4.17% and 30% females, respectively. When considering them as RR + HF cohort, the mean age was 59 ± 14 years and 15.91% were females. Each model consists of left and right ventricle tetrahedral meshes derived from four-chambers models (Fig. 1). Each mesh includes universal ventricular coordinates (UVC) (J. Bayer et al., 2018) and rule-based fibres (J. D. Bayer et al., 2012).

### 2.2 Lead designs

Lead designs were assumed to have 4 electrodes spaced along them. Inter-electrode distances in current quadripole leads vary between 7.5 and 35.5 mm depending on the vendor, with a maximum distance from the proximal to the distal electrode of 60 mm in the case of the model L from Biotronik [7]. To capture this variability, 8 possible electrode positions were considered spread over 52.5 mm with an inter-electrode distance of 7.5 mm. Some leads include a bend to increase stability, but this scenario was not included in the simulations.

**Fig. 2:**
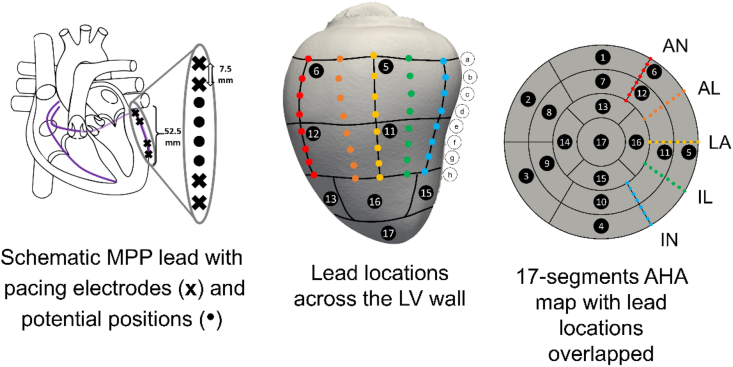
Schematic positioning of the LV leads. Each colour corresponds to a possible vein location in the LV free wall, with a virtual lead placed in it. Each dot corresponds with a potential electrode location along the lead. AN stands for anterior, AL for antero-lateral, LA for lateral, IL for infero-lateral and IN for inferior. (For interpretation of the references to colour in this figure legend, the reader is referred to the Web version of this article.)

Virtual leads were placed in an idealised vertical straight configuration following five possible vein locations along the LV free wall, see Fig. 2. A personalised 17-segments AHA map was created for each patient using the UVC to define the 5 vein locations: at the border between AHA segments 4 and 5 referred to as inferior (IN); in the centre of AHA segment 5, inferolateral (IL); at the border between AHA segments 5 and 6, lateral (LA); in the centre of AHA segment 6, anterolateral (AL) and at the border between AHA segments 6 and 1, anterior (AN). The most basal electrode was set at 80% of the apicobasal distance and the remaining electrodes are in a line that follows the epicardium down to the apex.

Throughout this study, the electrodes within each lead are named with a letter indicating their position. The most basal electrode corresponds to letter “a”, while the most apical electrode corresponds to letter “h”. When more than one electrode has been paced at a time, the names of the electrodes activated are appended up to a total of four. For instance, activation “ad” corresponds to the simultaneous activation of electrodes “a” (closest to the base) and “d” (at 22.5 mm from the base). Since there are only eight electrodes in each lead, if a configuration has more than one letter (such as “ad”) this indicates that more than one electrode is activated.

An extra electrode on a separate lead was used in all the cases, placed on the endocardium of the RV apex. Pacing from the RV lead alone served as a baseline simulation that approximated the effect of left bundle branch block activation. This simulation was used as a reference for evaluating the effect of LV pacing.

For MPP pacing, one or two electrodes on the LV were stimulated simultaneously with the RV electrode, depending on the configuration that is being evaluated. Stimulations approximated an LV lead to RV coil vector so that the activation started at the electrode site. Other pacing vectors were not considered.

### 2.3 Electrophysiology simulations

Electrophysiology simulations were run using the reaction-eikonal model [17]. The conduction velocity (CV) of the myocardium in the fibre direction was set to $CV_{fibre}=0.5$ m/s and the normal and transversal fibre direction CV was set to 60% of $CV_{fibre}$ $\left(k_{xf}=0.6\right)$ based on literature values [18]. To simulate the rapid activation of the ventricular endocardium to obtain physiological results, an isotropic fast endocardial conduction (FEC) layer one-element thick was added to the bottom third of the endocardium [19]. The CV in this layer was set to be proportional to the $CV_{fibre}$ with a factor of $k_{FEC}=5$, based on computational studies [20].

The total activation time of both ventricles (TAT) was calculated as an approximation of QRS duration. To avoid the spurious effects in TAT caused by small remote myocardial segments, the metric considered was the time taken to activate 90% of the volume of the biventricular myocardium (AT090). The benefit of pacing was calculated as the normalised change relative to the value achieved with RV pacing.

To test if anatomical remodelling impacts optimal lead design, independent of any cellular remodelling, we compare the HF subjects to RR models using the same material parameters. As we did not tune the models to data from individual patients, the models were not validated individually. The validation was performed in a cohort-based manner, comparing metrics extracted from the cohort against values from corresponding cohorts in the literature.

### 2.4 Choice of optimal lead designs

In this work, we will refer to the combination of four electrodes as a “lead” or “lead design”. Within each lead, the specific electrodes activated will be referred to as “lead configuration”.

A clustering technique, Hierarchical Agglomerative Clustering (HAC) [21], was used to identify groups of patients who had a similar response to similar lead designs and identify lead designs that benefited the most patients. The distance between clusters was based on how similar were the activation for a given patient or a given design using the Euclidean distance as the clustering distance and the Ward2 criterion as the clustering criterion [[22], [23], [24]]. More details on this technique can be found in Supplement 1.

All 1 and 2-electrode permutations from the 8 electrode positions were evaluated, leading to 70 combinations that were evaluated in 5 potential vein locations (AN, AL, LA, IL, IN) in three cohorts (RR, HF and RR + HF), leading to a total of n=70×5×44=15400 simulations. Although technically there are two cohorts and a combined group, we will refer to them as three cohorts to ease the readability of the results.

Two definitions of optimal designs are used: first, personalised (or patient-based) lead designs, where the design is tailored for each patient and each vein; and second, cohort-based (or population-based, analogous to HF-based, RR-based or RR + HF-based) optimal lead designs, where the design is chosen for the whole cohort, assuming that it is placed in the best vein position for each patient.

**Fig. 3:**
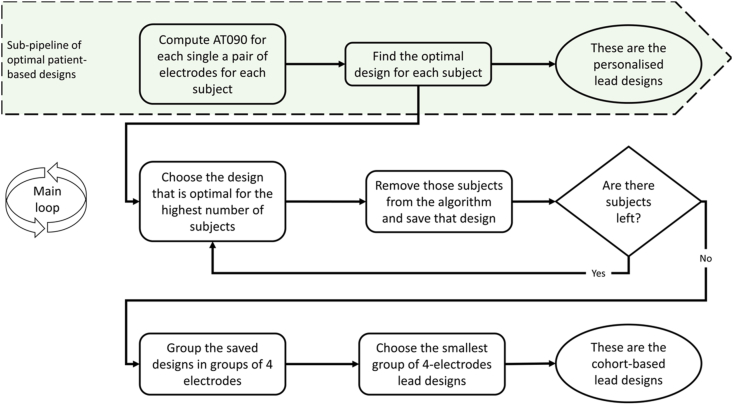
Schematic diagram of the pipeline followed to find the patient-based and cohort-based designs.

The patient-based optimal quadripolar lead design was defined as the one that maximises the response (i.e. reduction of AT090). In the case of cohort-based, the criterion is to maximise the number of subjects that achieve the optimal (i.e. maximal) response. This outputs choices of single and pairs of electrodes. These are combined in groups of four electrodes (creating a lead), with the criterion of having the minimum number of (four-electrode) lead designs needed to cover all optimal electrodes configurations. This pipeline is shown in the schematic Fig. 3. The first row corresponds to the steps necessary to achieve the patient-based optimal designs, while the cohort-based designs are outputted at the end of the diagram following the direction of the arrows.

Once the HF-based optimal quadripolar lead design was found, the benefit from further tailoring the activated electrodes within the lead to each patient and vein was investigated. The same comparison was done in the RR cohort. Lastly, the performance of the cohort-based optimal lead designs was compared against each other using the HF-based lead design in the RR cohort and vice versa.

### 2.5 Sensitivity analysis

The impact of uncertainty in the model due to these main parameters was estimated by repeating the simulations varying one parameter at a time to 2 different values. All the parameters were set to the literature values reported earlier in the text, except for one each time. The FEC layer size was varied from 33% to 70% and 100% (whole endocardium); $CV_{fibre}$ was varied from 0.5 to 0.07 and 0.8 m/s; the fibre anisotropy $k_{xf}$ was varied from 0.6 to 0.29 and 1, and the endocardial anisotropy $k_{FEC}$ was varied from 5 to 7 and 10.

As baseline activation, RV apical pacing was used to approximate physiological activation under left bundle branch block. To assess the relevance of the position of the RV lead, this pacing location was modified to the septal mid-wall using the AHA segments and the simulations were repeated.

CT images do not include any information about the level of scar present. To account for this, a first-order approximation of the effect of scarred tissue was introduced by blocking the conductivity when the LV wall is thinner than 5 mm [25,26].

### 2.6 Statistical analysis

Throughout this study, if it is not stated otherwise, the relative (in %) and absolute (in ms) reduction in activation time achieved with LV pacing compared to the RV pacing as a baseline are reported.

To assess the statistical significance when pacing in the LV wall with 1 or 2 electrodes, Wilcoxon signed-rank tests [27] were used with the relative reduction for each pacing situation in each of the hearts. The null hypothesis is that the distribution is symmetric about μ=0 and the alternative hypothesis was $H_{a}:\mu >0$, where μ is the location shift.

When comparing a cohort-based against the patient-based optimal lead designs, a Mann-Whitney test [28] (equivalent to unpaired Wilcoxon signed-rank test) was used with the null hypothesis that the distribution of the reduction of both pacing strategies does not differ and the alternative hypothesis that the personalised optimal lead designs have a greater reduction than the design given.

Similarly, to compare cohort-based designs and patient-based designs in both HF and RR cohorts, pair-wise Mann-Whitney tests were used with the alternative hypothesis that the two distributions compared do differ by a shift μ>0. Bonferroni corrections [29] were applied to avoid spurious positives due to multiple tests. The same strategy was used to compare cohort-based optimal lead designs in both RR and HF cohorts.

In all the cases a result was considered significant when p<0.01 and not significant otherwise. If not stated otherwise, the analyses were done on the relative reduction in AT090 since the absolute results (in ms) could be misleading due to the size of some hearts, especially in the HF cohort. All the statistical analyses were done in R using the native package stats [30].

## 3 Results

### 3.1 Models are representative of the expected QRSd

Average simulated TATs (Table 1) were compared against reported QRSd clinical measurements. In the case of the HF cohort, TATs were 167.28 ± 18.77 ms, being similar to reported values of 162 ± 16 ms [31] and 173.9 ± 32.8 ms [32]. With the same parameters, TATs for the RR cohort (137.66 ± 10.26 ms) matched QRSd of CRT responders of 133 ± 23 ms [33] and 141 ± 19 ms [34].

**Table 1:** Total activation time (TAT) for each one of the patients in the RR and HF cohort.

RR subject	TAT (ms)	HF subject	TAT (ms)
01	138.23	**01**	139.09
02	146.21	**02**	194.78
03	147.54	**03**	164.52
04	152.98	**04**	170.74
05	120.78	**05**	200.80
06	119.80	**06**	176.99
07	124.21	**07**	160.79
08	140.39	**08**	166.03
09	129.24	**09**	165.40
10	141.13	**10**	158.23
11	143.32	**11**	155.76
12	129.41	**12**	169.38
13	149.69	**13**	170.91
14	154.07	**14**	210.03
15	130.43	**15**	192.81
16	140.38	**16**	148.42
17	144.50	**17**	165.14
18	128.26	**18**	140.33
19	134.52	**19**	190.16
20	138.00	**20**	163.33
		**21**	179.57
		**22**	167.43
		**23**	143.95
		**24**	150.61
**Total**	137.66 ± 10.26		167.28 ± 18.77

### 3.2 Measuring electrical synchrony

LV pacing in the HF cohort with 1 and 2 electrodes (in all the possible lead locations) reduced TAT (p < 0.01) by 13.04 ± 9.05 ms (7.54 ± 4.92%) with maximum values of 41.28 ms (22.99%) compared to a reduction in AT090 (p < 0.01) of 14.48 ± 5.01 ms (11.92 ± 3.51%) with maximum values of 32.29 ms (25.69%). TAT was less sensitive than AT090 to the addition of an LV pacing lead, with no change in TAT in 13% of cases with the addition of an LV lead. This is because, in this 13% of HF cases (up to 65% of the cases in the RR cohort), the latest activated region was in the RV outflow tract and the addition of an LV pacing electrode, while increasing bulk activation synchrony did not decrease TAT as the RV outflow tract remain activated from a wave initiated at the RV electrode site. AT090 was therefore used as the index of synchrony for further analysis.

Does heart's shape or electrode position change activation times in HF patients?

The HAC algorithm was applied to the simulations when pacing from one or two electrodes in the HF cohort for the five vein locations. HAC identified four main clusters of lead sets: one with a single design (design “gh”, the most apical), the second and biggest with at least one basal electrode (designs including electrodes letters “a”, “b” or “c”), and two mixed groups of mid-wall electrodes. These groups were consistent across the five different vein locations. No consistent pattern was found, being the clustering different for the vein location except for the case HF21 (the one with the greatest response).

Although no specific anatomical feature was found to be driving the results, we found a higher variance in the reduction of AT090 across the different anatomies than across the different lead designs: the mean reduction in AT090 per patient (i.e. averaging all the electrode combinations in the LA vein) spanned from 8.7 to 30.89 ms (8.81%–24.58%) - in contrast, the mean reduction per combination of electrodes in the LA vein (i.e. averaging all patients) spanned from 9.48 to 16.87 ms (7.6%–13.91%). These findings were qualitatively consistent across the five lead locations, not only the LA vein. These results suggest that the major determinant of reduction in activation time is patient anatomy.

### 3.3 Optimal quadripolar lead designs for each cohort

**Fig. 4:**
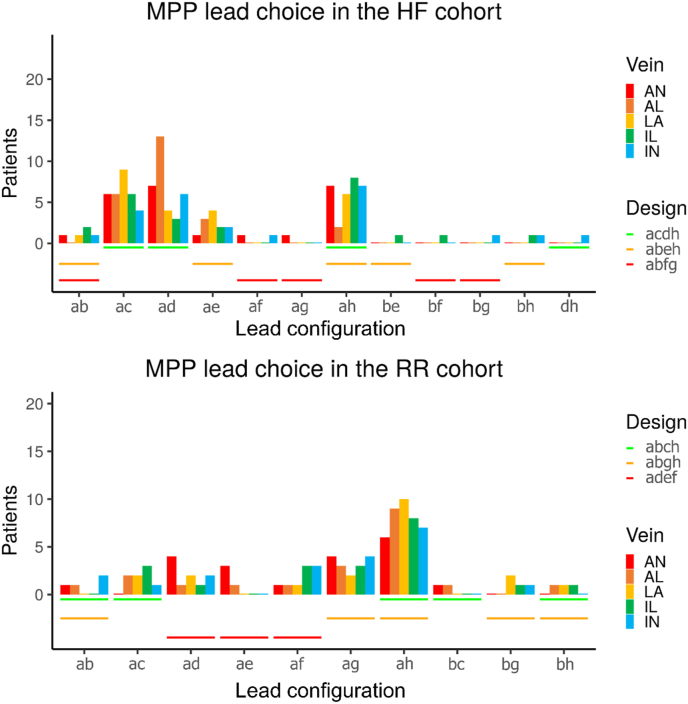
Optimal MPP design across all veins. The order followed was first choosing the ones where the maximum number of patients are benefited from it while using the minimal amount of lead designs.

Lead design “acdh” was the HF-based optimal lead design (see Fig. 4, top). Only three lead designs were needed to achieve an optimal response (as defined in the Methods section) in each patient in the HF cohort and a different set of three designs for the RR cohort. In both cohorts, the lead configuration “ah” (electrodes maximally spaced) was one of the most effective in most of the patients. In all but one case (configuration “dh”), all the configurations included at least one basal electrode (electrode “a” or “b”).

The main difference between HF and RR cohorts was that apical electrodes were more effective in the RR cohort, while mid-wall electrodes were the most effective in the HF cohort after basal configurations (i.e. see the prominence of “ac” and “ad” configurations in HF in Fig. 4). This may be due to differences in the size or shape of the hearts. To assess the impact of LV size (bigger in the HF cohort than in the RR cohort), we increased the size of the meshes of the RR cohort until the LV volume matched the average LV volume of the HF cohort (more details in S2). Although the optimal design changed slightly, the trend of being less mid-wall compared to the HF cohort persist (i.e. “ac” and “ad” configurations are not prominent in the enlarged RR cohort), indicating that the differences were due to shape, such as the wall thickness or the curvature of the ventricles.

### 3.4 Effect of anatomical remodelling on optimal quadripolar lead design

To evaluate how anatomical remodelling altered the predicted response to MPP, the distribution of change in activation time with all lead designs in the RR and HF cohorts was compared.

**Fig. 5:**
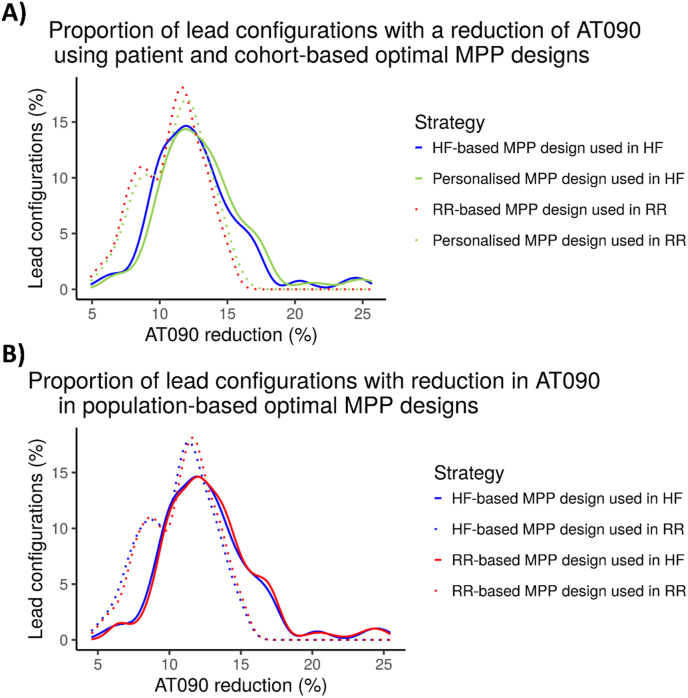
Distributions of AT090 reduction (in %) for different lead configurations in each of the cohorts. The distributions correspond to kernel density estimates from discrete data points. A) Proportion of lead configurations with a reduction of AT090 using personalised and population-based optimal quadripolar lead designs. B) All the options of single optimal lead design for each cohort applied to each cohort. Colour corresponds to the cohort for which the quadripolar lead is optimal and solid or dotted corresponds to the cohort where the quadripolar lead is applied to. (For interpretation of the references to colour in this figure legend, the reader is referred to the Web version of this article.)

The mean AT090 reduction was smaller (p < 0.01) in the RR cohort than in the HF cohort, see Fig. 5A. Compared to the cohort-based optimal design, a personalised optimal MPP design improved AT090 reduction non-significantly from a mean of 12.68%–13.16% (p > 0.01) in the HF cohort. Analogously, in the RR cohort, personalising the design non-significantly reduced AT090 from a mean of 10.81%–11.13% (p > 0.01).

In the case of using the RR-based quadripolar lead design in the HF cohort, the mean reduction was 12.85% (see Fig. 5B). Although this value was greater than the mean reduction using the HF-based lead design (12.68%), it was not statistically significant (p > 0.01). With the RR cohort, if using the HF-based optimal lead design, a reduction in AT090 of 10.62% was achieved, not being statistically different from using the RR-based optimal lead design (p > 0.01, mean value of 10.81%).

### 3.5 One-at-a-time sensitivity analysis

The virtual patient cohorts implicitly encode several modelling assumptions and best estimates of model parameters. To test if any of these assumptions impacted the overall conclusions, a systematic sensitivity analysis was performed (details in Supplement 2).

The parameters related to the Purkinje network modelling (FEC layer size and $k_{FEC}$) were the main parameters that impacted the study conclusions. In general, the larger the conductivity (larger size or faster $k_{FEC}$) was, the more basal the electrodes were chosen for optimal lead design, with the most extreme lead design of “abcd” for $k_{FEC}=10$ in the RR + HF cohort. These results indicate that the conductivity and size of the Purkinje network may impact optimal lead design.

In general, the results did not change when the rest of the parameters were changed. The optimal lead configuration was consistently found to be a basal and apical electrode (typically “a” and “h”) combined with two mid-wall electrodes.

## 4 Discussion

We performed an in-silico study to estimate the benefit of patient-specific lead designs for achieving electrical resynchronization in an HF and RR cohort. We found that pacing with a single (cohort-based) lead design is not significantly different from pacing with personalised lead designs if no scar is taken into account.

Computational models were used to isolate the effect of the anatomy, creating a virtual cohort study that would have been unfeasible in a clinical trial due to all the possible combinations of lead designs and locations. Although this work was not a longitudinal study, using healthy anatomies as approximations of RR hearts revealed that anatomies that approximate RR hearts respond in a different way to MPP compared to HF anatomies.

MPP leads offer the ability to personalize pacing protocols to individual patients. As each lead can deliver multiple programable pacing vectors, the therapy can be adjusted over time, potentially adjusting to changes in the patient's heart as they reverse remodel. There are many different lead designs provided by different vendors, providing clinicians with some choice on the distribution of electrodes that they can implant in any one patient. In this study, we aimed to test, using simulations, if there is evidence, based on patient anatomy, to support the selection of specific leads to individual patients and if the lead design is best initially likely to remain optimal if the heart reverse remodels. Our study found that a single lead design was likely to provide near-optimal activation synchrony initially and after remodelling and that the ability to reprogram vectors provided sufficient personalization without the need for patient-specific lead designs. However, while we estimated the impact of scar, we were not able to directly image scar for each patient and including the effects of scar may impact these results. Impact of anatomy in lead design and lead configurations.

The HAC algorithm showed that there exists a difference in response due to anatomy. This finding explains partially the percentages of non-responders [3] since patients with anatomy closer to RR have a significantly lower response compared to HF anatomy (Fig. 5).

In this work, reverse remodelling was restricted to the organ 3D anatomy, ignoring functional remodelling by fixing all the model material parameters that encode patent electrophysiological function. Although functional remodelling has been widely documented, we did not have data to inform changes in model parameters. However, while we did use the same parameters for both HF and RR cohorts, we still recovered QRS durations typical for HF patients and CRT responders, respectively. To understand better this change in QRS duration, we tested two additional scenarios. In the first scenario, we reduced the size of the HF meshes to 75% their size to match the RR mean volume (more details in Supplement S2). This resulted in a mean QRS duration of 127 ± 14 ms, even smaller than the average 138 ± 10 ms of the RR cohort. In the second scenario, we kept the HF size, but we used parameter values typical of a healthy subject and observed a reduction as well in QRS duration to 78 ± 11 ms (more details in Supplement S2). Considering these results, we can say that a change in parameters could have a bigger effect than a change in size for the reduction of QRS duration, but the smaller size of the hearts in the case of the RR cohort is sufficient to explain the shorter QRS durations reported in clinical studies [33].

When comparing the cohort-based optimal quadripolar lead designs applied to both cohorts, the distribution of lead configurations was shown to be more dependent on the cohort where the lead was applied, rather than on the specific design itself (see Fig. 5B). These findings together with the results obtained in the HAC algorithm, where more disparity was found in patients’ difference than leads configurations difference, suggest that anatomical differences are key for selecting the correct vein locations and electrodes to stimulate but have a limited impact on the lead design. This is in line with the findings of patient selection being a key criterion to obtain optimal results [35,36].

In this work, the main metric to measure electrical dyssynchrony has been AT090, using one depolarization wave. We used computer models to simulate this time, as opposed to calculating geodesic distances to estimate activation time, as the models can account for spatially varying orthotropic conductivity due to the fibre microstructure, and the FEC layer, which has been found to impact to have a major impact on activation patterns and CRT response [20].

### 4.1 Using virtual populations as pilot studies

Comparing the effectiveness of multiple lead designs is a task generally unfeasible in clinical studies due to the sample size required to obtain statistically significant results, and time to compare all the possible different lead designs. Methodologies such as network meta-analysis [11,12] could be used to analyse the designs compared in several studies against a common reference, such as no device implantation. Computational modelling and simulations offer a different, less costly strategy to tackle this problem.

In this work, virtual 3D anatomies and electrophysiology simulations were used to study lead designs in two patient groups across five possible vein locations, for a total of over 15,000 simulations. As the complexity of single devices is increased, the permutations of devices implanted in a single patient also increase [37,38] as will the range of settings, device combinations, and implant locations. To deal with this increased sampling space, simulations will have a growing role in cardiac device design [14].

This work shows the ability to perform a study on lead designs informed from designs across multiple vendors in a common patient group. This framework allows designs to be compared, new designs evaluated and new optimal designs proposed using a digital twin approach [39]. Although more clinical studies will be needed to validate these results, this study demonstrates how virtual trials can be a useful tool for exploratory or pilot studies.

### 4.2 Usage of MPP leads over conventional CRT leads

One of the main disadvantages of MPP leads against conventional CRT devices is that the increased number of activated electrodes in the same lead can reduce considerably their battery life. Although there is not extensive literature on this, the latest estimations suggest a reduction of the battery longevity by approximately 15% [40]. Therefore, they should only be implanted if there is sufficient evidence of an improvement (or non-deterioration) of the patient's prognosis.

According to the largest systematic review and meta-analysis on the use of MPP up to date [41], there is not enough solid evidence yet to prefer MPP over conventional CRT device or vice versa in all the patients. When evaluating both randomised and nonrandomised studies together, MPP demonstrates superiority over conventional CRT, although this improvement is lost when considering randomised studies. In the studies analysed, the populations and end points were considerably heterogeneous and conclusive statements about the therapeutic benefit of MPP is not straightforward to get.

In our study, we found that the optimal activation of 2 electrodes in the HF cohort causes a reduction of AT090 of 14.52 ± 3.25%. Considering the mean activation of a single electrode – analogous to a conventional CRT pacing – the reduction of AT090 is 10.91 ± 2.77%. On average, the MPP optimal configuration is thus multiplying the reduction of AT090 with a factor of 1.33.

We notice that even if on average we achieve a modest improvement over a conventional CRT configuration, for specific patients it can be enough to justify the higher consumption of battery of these devices.

## 5 Limitations

This work makes use of simulation and modelling steps, and results should be understood and interpreted within this framework. Several limitations are noted in this regard.

The anatomy of the coronary sinus of each one of the cases was not included. Although in clinical studies the exact vein position and shape is essential to anchor the leads, a generalised straight line was used in five different positions across the LV wall to model the effect of the coronary sinus. Although in some particular cases this simplification might suppose a qualitative difference in the results, the effect of pacing in different vein positions is reflected in the range of vein locations evaluated.

The results of this work are based upon the choice of certain critical parameters for the modelling in simulations. Although the cohorts have been validated by comparing them with results in the literature, patient-specific functional was not validated. In the one-at-a-time sensitivity analysis, two extra values were tested for each one of the most relevant parameters. The FEC layer was shown to be a key element in the simulations. This element approximates the Purkinje system and depending on the patient there might be bigger differences in the size and conductivity of this network. However, while the FEC layer did impact simulations it did not change the overall study conclusions.

To be able to isolate the role of anatomy in the results, the same material properties were used in all the cases. Although the QRS durations obtained are comparable with the values found in the literature, there could be subjects with modified properties, such as a higher CV in the RR cohort. We tested this by adding a case in the SA, where we used conductivity parameter values derived from healthy subjects (more details in Supplement S2). This resulted in a QRS duration for the RR cohort of 65 ± 21 ms, too low compared to the reported clinical durations in CRT-responders patients of 133 ± 23 ms. Regarding the rest of the parameters in the (local) sensitivity analysis, we observed that the changes are in general negligible except for the parameters affecting the FEC layer. Results should be interpreted considering the range of modelling scenarios that lacked personalised EP parameters. Scar tissue was not available through the CT images. For the sensitivity analysis, the thinnest regions (<5 mm) in the HF cohort were set as non-conductive. This is a first-order modelling approximation of scarred tissue, based on the work of Takigawa et al. [42] and only provides an approximation of the clinical effect of scar tissue on the results. To assess the impact of this modelling assumption we added an extra case in the sensitivity analysis modelling the scar tissue as those regions with a thickness < 6 mm. In general, there is not a qualitative different between the 5 mm and 6 mm scenarios, except that in the second scenario, the difference between pacing with a personalised MPP lead and a cohort-based optimised design is greater (more details in Supplement S2). More research would be needed using models that include personalised scar information derived from images [43].

The size of the LV plays an important role in CRT response, as shown previously by other researchers [44,45]. This factor is implicitly included when separating between HF cases and RR cases, since HF are bigger than when the heart remodels back to health. In Fig. 1 in S1, we can see the LV volumes of each HF case in the clustering, showing no clear pattern for bigger or small size linked to the lead configurations. This is due probably due to the small sample size, rather than a lack of relation between LV volume and CRT response. Pacing in the most basal and most apical positions was found effective in this study with a 50–60 mm distance between the electrodes, however, this configuration is not present in the commercially available designs enumerated by Antoniadis et al. [7]. Nonetheless, specific vein anatomy was not taken into consideration in this study, as well as difficulties with lead implantations. These elements must be taken into account in clinical practice, but they have a limited impact on current in-silico studies.

The use of statistical significance tests can be problematic in simulation studies. As described by White et al. [46], there are two main reasons for this. First, the sample size is one of the main drivers of statistical significance. In our case, having more than 15000 simulations, is very likely that the p-values obtained are smaller than virtually any arbitrary threshold established. Second, there is a more philosophical question of the validity of the null hypothesis in in-silico experiments. In these cases, it is known a priori that the null hypothesis of no difference between different treatments (pacing sites in our case) is false, therefore compromising the validity of the test. As is noted by White et al., “the question is not whether the model outcomes will be different, but rather how different they will be”. As is shown in Fig. 5 and mentioned throughout the text, we show the magnitude of the differences between different pacing protocols, regardless of specific p-values.

## 6 Conclusion

Pacing with a single (optimised) lead design is not qualitatively different from pacing with personalised lead designs. Either with personalised lead designs or with a single lead, the response in chronic reverse remodelled anatomies is qualitatively different than in the acute situation in heart failure anatomies. This study shows the potential of virtual clinical trials as tools to explore new lead designs.

## Author contributions

Conceptualization: Cristobal Rodero, Pablo Lamata, Steven A. Niederer.

Data curation: Christopher A. Rinaldi, Steven A. Niederer.

Formal analysis: Cristobal Rodero.

Funding acquisition: Pablo Lamata, Steven A. Niederer.

Investigation: Cristobal Rodero, Marina Strocchi, Angela W.C. Lee, Pablo Lamata, Steven A. Niederer.

Methodology: Cristobal Rodero, Marina Strocchi, Angela W.C. Lee, Pablo Lamata, Steven A. Niederer.

Project administration: Pablo Lamata, Steven A. Niederer.

Resources: Christopher A. Rinaldi, Pablo Lamata, Steven A. Niederer.

Software: Gernot Plank, Edward J. Vigmond.

Supervision: Pablo Lamata, Steven A. Niederer.

Visualization: Cristobal Rodero.

Writing – original draft: Cristobal Rodero.

Writing – review & editing: Marina Strocchi Angela W.C. Lee, Christopher A. Rinaldi, Gernot Plank, Edward J. Vigmond, Pablo Lamata, Steven A. Niederer.

## Funding

CR received funding from the European Union's 10.13039/501100007601Horizon 2020 Research and innovation programme under the Marie Sklodowska-Curie Grant Agreement No 764738.

MS was supported by an unrestricted Abbott educational grant through the Centre for Doctoral Training in Medical Imaging at King's College London.

EJV was supported by the French Government as part of the “Investments of the Future” program managed by the National Research Agency (ANR), Grant reference ANR-10-IAHU-04.

GP received support from the Austrian Science Fund (10.13039/501100002428FWF) (https://fwf.ac.at/en/); grant number PI2760–B30 and the EU (MedalCare 18HLT07).

PL is supported by 10.13039/501100000274BHF [PG/16/75/32383] and holds a 10.13039/100010269Wellcome Trust Senior Research Fellowship [WT 209450/Z/17/Z].

SAN is supported by NIH R01-HL152256, ERC PREDICT-HF 453 (864055), 10.13039/501100000274BHF (RG/20/4/34803), 10.13039/501100000266EPSRC (EP/P01268X/1).

This work was supported by the 10.13039/100010269Wellcome Trust/10.13039/501100000266EPSRC Centre for Medical Engineering [WT 203148/Z/16/Z].

The funders had no role in study design, data collection and analysis, decision to publish, or preparation of the manuscript.

## Declaration of competing interest

None declared.
