# Supplementary material for: Impact of anatomical reverse remodelling in the design of optimal quadripolar pacing leads: A computational study
Source: Comput Biol Med. 2022 Jan;140:105073. doi: 10.1016/j.compbiomed.2021.105073 (PMC8752960; doi:10.1016/j.compbiomed.2021.105073)
Supplement: Multimedia component 1 [file mmc1.docx]

Supplement 1 – Hierarchical agglomerative clustering

A supervised machine learning technique was used to tackle the amount of data analysed and to get a visual inspection of the impact of each one of the lead designs in each one of the hearts. Hierarchical Agglomerative Clustering (HAC) algorithms^1–3^ start by taking each data point (activation times in our case) as a single group, or cluster. According to a certain distance metric (clustering distance), the two closest clusters are then merged into a new cluster. This merging also depends on the linkage method (or clustering criterion) since if the cluster is not a single point, the minimal distance to the cluster cannot be defined uniquely. This process iterates until all the data is agglomerated in one single cluster.

A static visualisation of this dynamic process is the dendrogram, a tree-shaped diagram showing the different clusters and the distance between them. Contrary to other clustering methods such as k-means, the optimal number of clusters is not automatically obtained but specified by the user. Nonetheless, using a dendrogram we can threshold perpendicularly to the direction of the tree through the root of the longest branch, getting a sensible number of clusters, although the best threshold will depend on the data analysed.

For each one of the lead positions and each one of the metrics analysed, we have used HAC to visualise how both the patients and the lead design cluster according to the different reduction of activation times with respect to the baseline (RV apex activation). HAC was performed with R^4^. Dendrograms were created with the package dendextend^5^ and sorted using the package dendsort^6^. The heatmaps to visualise the results were created with the package ComplexHeatmap^7^.

The clusters for each one of the cases were decided to ease the visual interpretation, although the number of clusters is a post-processing parameter. The Euclidean distance was chosen as clustering distance due to its popularity and a lack of rationale for the distance between the points. For the clustering criterion, the Ward2 criterion was used, a modification of Ward’s criterion^8–10^. In this criterion, the function to be minimised in each iteration is the change in variance, so more homogeneous clusters are achieved.

In Figure 1 we show the HAC maps for each one of the veins. On the left of the plot, the dendrograms clustering the lead designs, and on top are the dendrograms clustering the HF cases. Below the top dendrogram we have added barplots with the LV volumes of each case. If the bar is dark blue it indicates that the volume for that subject is higher than the average value (269 mL), while if it is light blue it indicates that the volume is smaller than the average of the cohort. Although it has been shown in previous studies^11,12^ we could not find any conclusive pattern that related the LV volume with the clustering of the cases. This is probably due to the small sample size of the HF cohort.


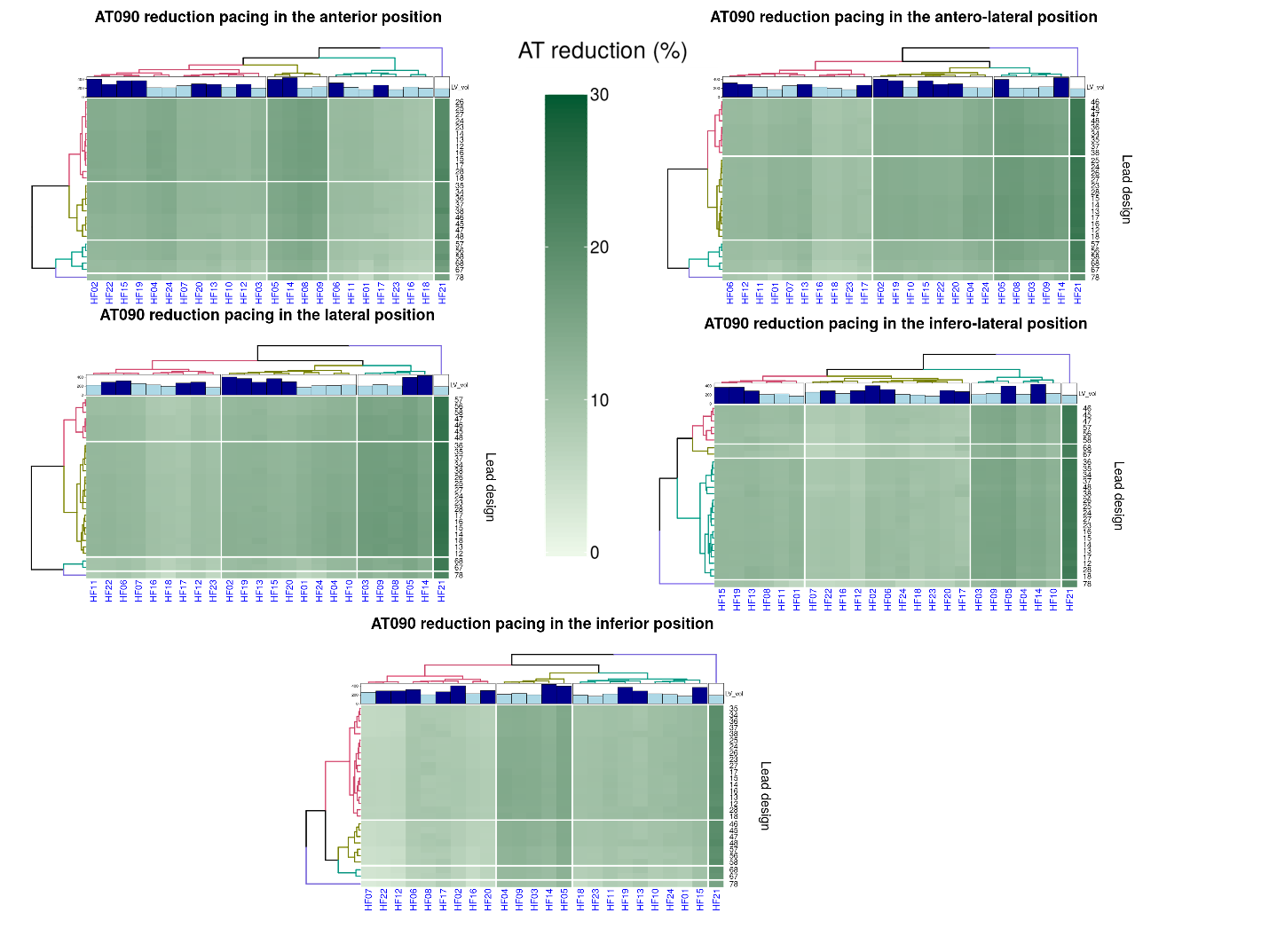


Figure 1 HAC plots for the different veins in the HF cohort. Barplots with the LV volumes are on top, where dark blue indicates volumes higher than the average volume of the cohort and light blue indicates volumes smaller than the average volume of the cohort.

# Bibliography

1. King B. Step-Wise Clustering Procedures. *J Am Stat Assoc*. 1967;62(317):86-101. doi:10.1080/01621459.1967.10482890

2. Murtagh F. A Survey of Recent Advances in Hierarchical Clustering Algorithms. *Comput J*. 1983;26(4):354-359. doi:10.1093/comjnl/26.4.354

3. Ward JH. Hierarchical Grouping to Optimize an Objective Function. *J Am Stat Assoc*. 1963;58(301):236-244. doi:10.1080/01621459.1963.10500845

4. R Core Team. R: a language and environment for statistical computing. https://www.r-project.org/. Published 2017. Accessed September 29, 2020.

5. Galili T. dendextend: an R package for visualizing, adjusting and comparing trees of hierarchical clustering. *Bioinformatics*. 2015;31(22):3718-3720. doi:10.1093/bioinformatics/btv428

6. Sakai R, Winand R, Verbeiren T, Moere A Vande, Aerts J. Dendsort: Modular leaf ordering methods for dendrogram representations in R. *F1000Research*. 2014;3:177. doi:10.12688/f1000research.4784.1

7. Gu Z, Eils R, Schlesner M. Complex heatmaps reveal patterns and correlations in multidimensional genomic data. *Bioinformatics*. 2016;32(18):2847-2849. doi:10.1093/bioinformatics/btw313

8. Kaufman L, Rousseeuw PJ. *Finding Groups in Data : An Introduction to Cluster Analysis - Partitioning Around Medoids (Program PAM)*. Wiley; 1990.

9. Legendre P, Legendre L. Numerical Ecology Ch 6 - Multidimensional qualitative data. *Dev Environ Model*. 2012;24:337-424. doi:10.1016/B978-0-444-53868-0.50008-3

10. Murtagh F, Legendre P. Ward’s Hierarchical Agglomerative Clustering Method: Which Algorithms Implement Ward’s Criterion? *J Classif*. 2014;31(3):274-295. doi:10.1007/s00357-014-9161-z

11. Zweerink A, De Roest GJ, Wu L, et al. Prediction of Acute Response to Cardiac Resynchronization Therapy by Means of the Misbalance in Regional Left Ventricular Myocardial Work. *J Card Fail*. 2016;22(2):133-142. doi:10.1016/J.CARDFAIL.2015.10.020

12. Varma N, Baker J, Tomassoni G, et al. Left Ventricular Enlargement, Cardiac Resynchronization Therapy Efficacy, and Impact of MultiPoint Pacing. *Circ Arrhythm Electrophysiol*. 2020;13(11):e008680. doi:10.1161/CIRCEP.120.008680
