# Supplementary material for: Impact of anatomical reverse remodelling in the design of optimal quadripolar pacing leads: A computational study
Source: Comput Biol Med. 2022 Jan;140:105073. doi: 10.1016/j.compbiomed.2021.105073 (PMC8752960; doi:10.1016/j.compbiomed.2021.105073)
Supplement: Multimedia component 2 [file mmc2.docx]

One-at-a-time sensitivity analysis

The mean reduction in AT090 when pacing with the personalised optimal lead design varied in the HF cohort from 13.16% in the default simulations to 15.92% when the FEC layer is extended to the whole endocardium; to 11.63% when $kFEC=10$ and to 14.56% when we set the myocardium to be isotropic ($k_{xf}=1$). In the case of the RR cohort, it varied (from 11.13%) in the case of an extended FEC layer to 70% of the apicobasal distance, to the whole endocardium and when $kFEC=10$ to 13.93%, 15.21% and 9.7% respectively.

The cohort-based optimal lead designs (”acdh”, “abch” and “abdh” in the default modelling for the HF, RR and RR+HF, respectively) change with most of the parameters modified as shown in Table 3.

| **Parameter** | **Value** | **HF-based design** | **RR-based design** | **RR+HF-based design** |
| --- | --- | --- | --- | --- |
| Default scenario | | acdh | abch | abdh |
| CV | 0.07 m/s | abdh | abch | abdh |
|  | 0.8 m/s | abdh | abfg | abdh |
| FEC layer extension | 70% apico-basal direction | abef | bcgh | abcf |
|  | Whole endocardium | aefh | abef | abfh |
| $k_{FEC}$ | 10 | abeg | abcd | abcd |
|  | 7 | acdh | abch | acdh |
| $k_{xf}$ | 0.29 | abde | abch | acdh |
|  | 1 | acdh | abch | adgh |

Table 1. Cohort-based optimal MPP lead designs when modifying different model parameters. CV stands for conduction velocity, FEC for fast endocardial conduction, kFEC for anisotropy in the FEC layer and kxf for cross-fibre anisotropy. Anisotropy ratios were defined based on the CV in the fibre direction.

The mean reduction in AT090 when using the cohort-based optimal lead design in the HF cohort changed from 12.68% to values ranging from 11.14% (when the FEC layer anisotropy is 10) to 14.38% (when the FEC layer covers the whole endocardium). Only when the FEC layer covers 70% of the apicobasal distance, we obtain a value not significantly different from the default set up. The same situation is present in the RR cohort.

We tested the effect of the parameters on the differences in AT090 reduction when pacing using a cohort-based optimal design or using a personalised optimal design (as shown in Figure 6A). The only parameter affecting this difference is the extension of the FEC layer. When the FEC layer is extended to 70% of the apicobasal distance, personalising the designs in the RR cohort increases the reduction in AT090 in a significant way compared to using the cohort-based design (13.93% and 12.23%, respectively). This difference is also present in the HF cohort, where personalising the designs achieves a 14.19% reduction of AT090 while using the cohort-based design achieves 12.09% . In the case of extending the FEC layer to the whole endocardium, personalising the lead design in the HF cohort shows a significant difference compared to using a cohort-based design (15.92% and 14.38%, respectively).

In the case of using the cohort-based optimal lead design for each cohort in both cohorts (Figure 6B in the manuscript) the parameter that has influence in the results is the FEC layer extension as well. In both cases tested, the four mean values are not different from each other .

# Modifying the RV pacing location and scar tissue

Throughout this work, the RV apex is used as a pacing point reference, measuring the reduction in AT090 with respect to the activation time pacing on the RV apex. The analysis was repeated pacing in the middle of the RV septum instead to assess the effect of this reference point. The mean reduction in AT090 when pacing with the personalised optimal lead design varied in the HF cohort from 13.16% in the default simulations to 15.76%. In the RR cohort, it varied from 11.13% to 13.75%.

The optimal lead design is changed in the RR cohort from “abch” to ”abgh”, and consequently in the RR+HF cohort from “abdh” to ”abcg”. When using this design, the mean reduction in AT090 was increased from 10.81% to 12.41%. In the HF cohort, although the design did not change, the mean reduction in AT090 when using it did change from 12.68% in the default scenario to 15.21% when pacing in the septum.

Pacing in the septum instead of in the RV apex, caused a significant difference between pacing with the personalised lead design or with the cohort-based in the RR cohort (13.75% and 12.41%, respectively). It did not cause any other differences in terms of significant differences in any of the other scenarios considered in Figure 6.

To account for the potential presence of scar tissue, we added two scenarios with scar tissue present, a first scenario wherethe LV wall regions thinner than 5 mm were set as non-conductive, and a second scenario where the threshold was 6 mm. Cases 13 and 21 were discarded since they were modelled with a constant thickness^1^. The mean reduction in AT090 when pacing with the personalised optimal lead design varied from 13.16% in the default simulations to 13.39% and 13.06% using the 5 and 6 mm scar threshold, respectively. The cohort-based optimal lead design changed from “acdh” in the default setup to “abdh” in both cases including scar and the mean reduction in AT090 when using it changed from 12.68% to 12.87% with the threshold of 5mm and to 12.13% with the threshold of 6mm.

Pacing with 5 mm scarred tissue, caused a difference between pacing with the personalised lead design or with the cohort-based (14.78% and 12.87%, respectively). In the 6 mm-threshold scenario the difference is even greater (14.91% for the personalised design and 12.14% for the cohort-based design).

# Modifying the size of the RR cohort

One of the main difference between HF and RR cohorts (with the default parameters as presented in the main text) was that apical electrodes were more effective in the RR cohort, while mid-wall electrodes were the most effective in the HF cohort after basal configurations. To assess if this could be attributed to the higher LV size (bigger in the HF cohort than in the RR cohort) we increased the size of the meshes of the RR cohort until the LV volume matched the average LV volume of the HF cohort.

Originally, the LV volume of the RR cohort was of 128±23 mL, and after a scaling of 1.25 it increased to 250±45 mL, comparable to that of the HF cohort of 269±78 mL. In Figure 1 we show the optimal lead designs with their distributions across the veins.


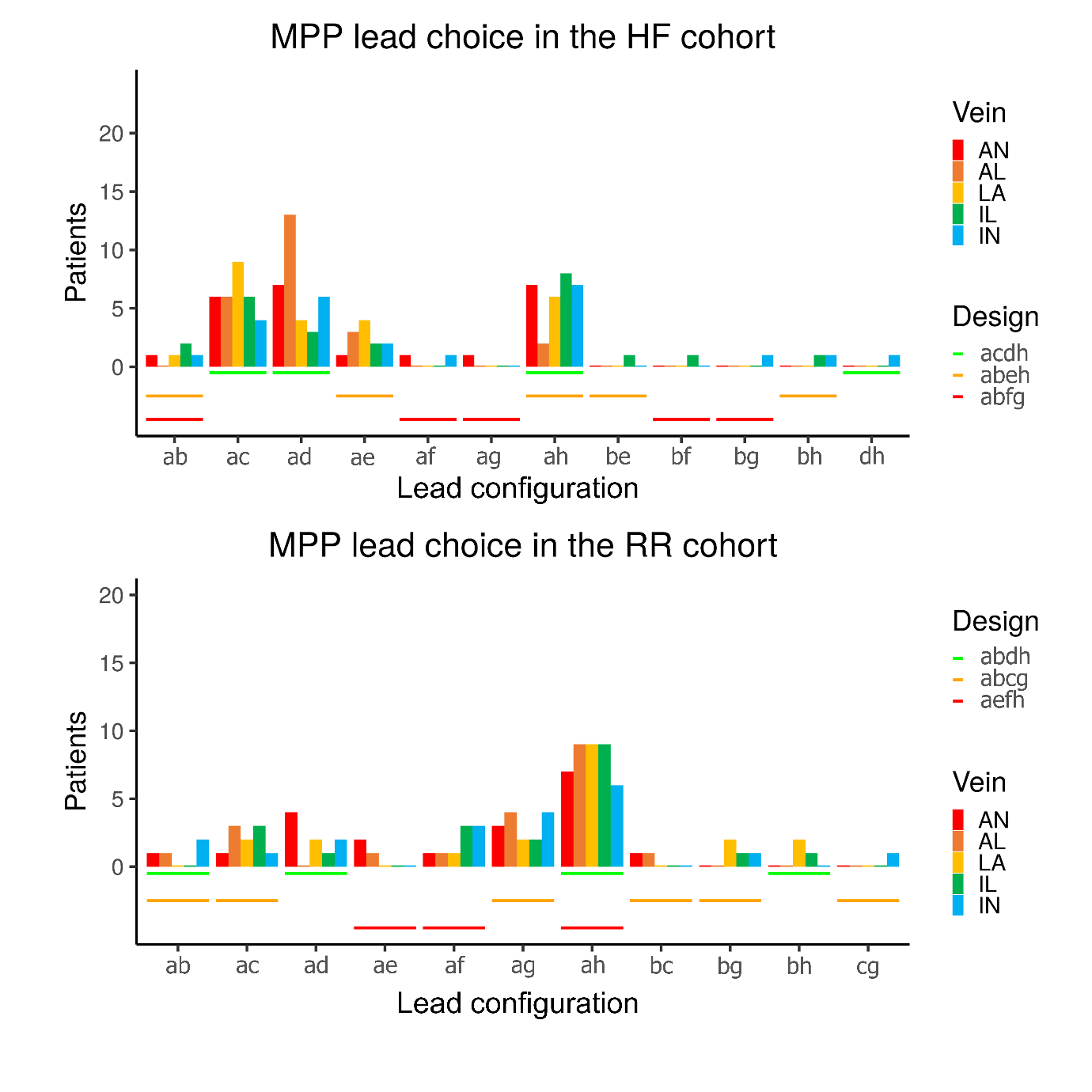


Figure 1. Optimal MPP design across all veins with an enlarged RR cohort. The order followed was first choosing the ones where the maximum number of patients are benefited from it while using the minimal amount of lead designs.

Although the optimal design changed slightly, the trend of being less mid-wall compared to the HF cohort persist. We can then only attribute it to local shape differences, such as the wall thickness or the curvature of the ventricles.

# Factors influencing QRS duration

In this work, reverse remodelling was restricted to the organ 3D anatomy, ignoring functional remodelling by fixing all the model material parameters that encode patent electrophysiological function. Although functional remodelling has been widely documented, we did not have data to inform changes in model parameters. However, while we did use the same parameters for both HF and RR cohorts, we still recovered QRS durations typical for HF patients and CRT responders, respectively. To understand better this change in QRS duration, we tested two additional scenarios.

In the first scenario, we reduced the size of the HF meshes. Originally, the LV volume of the HF cohort was 269±78 mL. After scaling them down with a factor of 0.75, we obtained an average LV volume of 113±32 mL, comparable to the RR cohort volume of 128±23 mL. This size reduction resulted in a mean QRS duration of 127±14 ms, even smaller than the average of 138 ± 10 ms of the RR cohort.

In the second scenario, we kept the HF size, but we used parameter values compatible with those of a healthy subject: a CV of 0.8 m/s, a cross-fibre anisotropy factor of 0.23, an endocardial anisotropy factor of 7 and a FEC height increased up to 70% of the distance from apex to base^2^, resulting in a QRS duration reduction down to 78±11 ms.

Considering these results, we can say that a change in parameters could have a bigger effect than a change in size for the reduction of QRS duration, but the fact of being smaller hearts in the case of the RR cohort is enough to achieve shorter QRS durations as reported in clinical studies^3^.

# Bibliography

1. Strocchi M, Augustin CM, Gsell MAF, et al. A publicly available virtual cohort of fourchamber heart meshes for cardiac electromechanics simulations. *PLoS One*. 2020;15(6 June):e0235145. doi:10.1371/journal.pone.0235145

2. Rodero C, Strocchi M, Marciniak M, et al. Linking statistical shape models and simulated function in the healthy adult human heart. Beard DA, ed. *PLOS Comput Biol*. 2021;17(4):e1008851. doi:10.1371/journal.pcbi.1008851

3. Bonakdar HR, Jorat MV, Fazelifar AF, et al. Prediction of response to cardiac resynchronization therapy using simple electrocardiographic and echocardiographic tools. *Europace*. 2009;11(10):1330-1337. doi:10.1093/europace/eup258
